# The green microalga *Lobosphaera incisa* harbours an arachidonate 15*S*‐lipoxygenase

**DOI:** 10.1111/plb.12920

**Published:** 2018-10-24

**Authors:** B. Djian, E. Hornung, T. Ischebeck, I. Feussner

**Affiliations:** ^1^ Department of Plant Biochemistry University of Goettingen Albrecht‐von‐Haller‐Institute for Plant Sciences Goettingen Germany; ^2^ Goettingen Metabolomics and Lipidomics Laboratory University of Goettingen Goettingen Center for Molecular Biosciences (GZMB) Goettingen Germany; ^3^ Department of Plant Biochemistry University of Goettingen Goettingen Center for Molecular Biosciences (GZMB) Goettingen Germany; ^4^ Department of Plant Biochemistry University of Goettingen International Center for Advanced Studies of Energy Conversion (ICASEC) Goettingen Germany

**Keywords:** Fatty acid hydroperoxide, lipid peroxidation, oxylipin formation, *Parietochloris incisa*, positional specificity, substrate orientation

## Abstract

- The green microalga *Lobosphaera incisa* is an oleaginous eukaryotic alga that is rich in arachidonic acid (20:4). Being rich in this polyunsaturated fatty acid (PUFA), however, makes it sensitive to oxidation. In plants, lipoxygenases (LOXs) are the major enzymes that oxidise these molecules.
- Here, we describe, to our best knowledge, the first characterisation of a cDNA encoding a LOX (LiLOX) from a green alga. To obtain first insights into its function, we expressed it in *E. coli*, purified the recombinant enzyme and analysed its enzyme activity.
- The protein sequence suggests that LiLOX and plastidic LOXs from bryophytes and flowering plants may share a common ancestor. The fact that LiLOX oxidises all PUFAs tested with a consistent oxidation on the carbon n‐6, suggests that PUFAs enter the substrate channel through their methyl group first (tail first). Additionally, LiLOX form the fatty acid hydroperoxide in strict *S* configuration.
- LiLOX may represent a good model to study plastid LOX, because it is stable after heterologous expression in *E. coli* and highly active *in vitro*. Moreover, as the first characterised LOX from green microalgae, it opens the possibility to study endogenous LOX pathways in these organisms.

## Introduction

Lipoxygenases (LOX) are non‐haeme iron enzymes and catalyse the oxidation of polyunsaturated fatty acids (PUFA). They play an important role in the synthesis of signalling molecules in animals (Brash 1999; Kühn *et al*. 2015) and plants (Wasternack & Feussner 2018; Wasternack & Hause 2013). Examples are leukotrienes in animals and jasmonates in plants (Brash 1999). LOX can produce a large number of hydroperoxy PUFA isomers, which may feed into different biosynthetic pathways. To meet the specific demands of these pathways, different LOX forms exist that are capable of oxidising PUFA with specific regio‐ and stereospecificity. In the simplest case, LOX oxidise PUFA harbouring at least one pentadiene system, as is the case for example in linoleic acid, a C18 PUFA with two double bonds (18:2(n‐6)). As a result, the hydroperoxy group can be found either at C‐9 or at C‐13. Consequently, LOX can be classified according to their regiospecificity either as linoleate 9‐ or 13‐LOX (Liavonchanka & Feussner 2006). The current models to explain their different regio‐ and stereospecificity can be divided in two: (i) the regiospecific abstraction of a specific hydrogen by catalytic iron, and (ii) the regio‐ and stereospecific attack of molecular oxygen at the substrate radical (Newcomer & Brash 2015; Schneider *et al*. 2007).

### Regiospecificity of hydrogen abstraction

**Figure 1:**
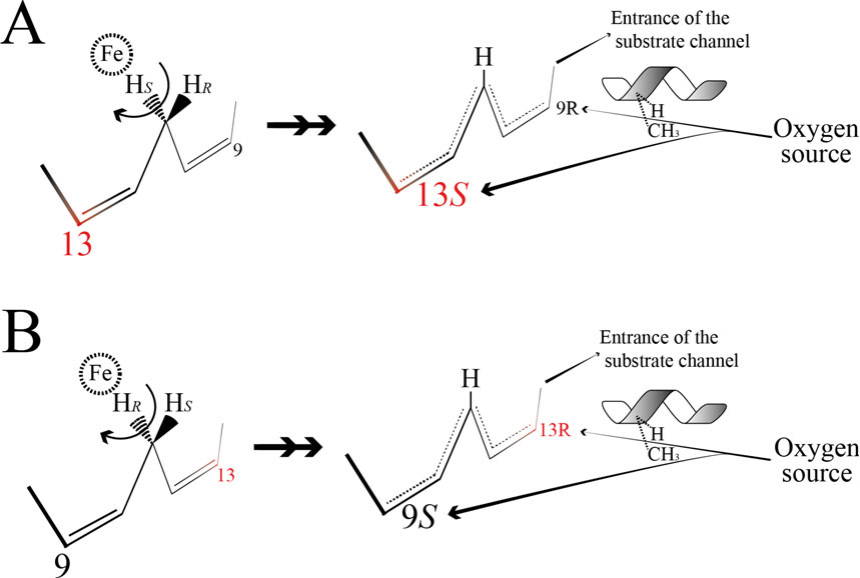
The LOX active site. In iron LOX, the catalytic metal is antarafacial to the oxygen source. The carbon 13 is represented in red. H and CH_3_ represent the Gly/Ala switch, described to influence the oxygen insertion. A: With tail‐first orientation, the Pro*S*‐Hydrogen is abstracted, typical for 13*S*‐LOX. B: With head‐to‐tail orientation, the Pro*R*‐Hydrogen is abstracted, typical for 9*S*‐LOX. Figure adapted from (Newcomer & Brash 2015).

The LOX rely on two mechanisms for homolytic C‐H bond cleavage to achieve specific hydrogen abstraction. First, the orientation of the substrate inside the LOX substrate channel. Either the methyl end is buried deep in the binding cavity (so called tail‐first) or the carboxyl group is inserted first (so‐called head‐to‐tail). The tail‐first or head‐to‐tail orientation changes the hydrogen presented to the catalytic iron, considering antarafacial dioxygen insertion (Fig. 1; Egmond *et al*. 1972; Hamberg & Samuelsson 1967). The feasibility of a PUFA inserting with head‐to‐tail orientation has been discussed because of the negative cost of burying a polar carboxyl group inside a non‐polar active site (Browner *et al*. 1998); this hypothesis may remain the best model available. It is supported by studies showing the formation of either 9*S*‐hydro(pero)xy octadecadienoic acid (H(P)ODE) or 13*S*‐H(P)ODE by the *Cucumis sativus* lipid body lipoxygenase (Cs‐LBLOX) after a point mutation (Hornung *et al*. 1999), as well as by *Momordica charantia* LOX1 (MoLOX1; Hornung *et al*. 2008) and *Glycine max* LOX1 (GmLOX1), showing a pH‐dependent product formation (Gardner 1989). Moreover, accepting two different orientations of the substrate makes it possible to explain the variability of positional oxidations of known iron‐LOX with a few variables. Further arguments in favour of this head‐to‐tail orientation have been summarised previously (Coffa *et al*. 2005b). Second, in the case of a substrate having more than one pentadiene system, the depth of substrate insertion (substrate frame‐shift) will influence the selection of the pentadiene system being attacked. This mechanism is attributed to the depth of the substrate channel, as shown experimentally for the human reticulocyte 15*S*‐LOX in the early 1990s (Sloane *et al*. 1991).

### Specificity of dioxygen attack

After abstraction of a specific hydrogen atom from the pentadiene system by a LOX, a substrate radical forms that delocalises throughout the newly formed conjugated pentadiene system. Next, formation of a hydroperoxyl radical can occur at two different carbon atoms (excluding *bis*‐allylic LOX) at either end of the pentadiene system, each with either *R* or *S* configuration (Fig. 1; Schneider *et al*. 2007).

### The pH may influence the regio‐ and stereospecificity

In addition, the pH of the reaction buffer influences the regio‐ and stereospecificity of some LOX (Gardner 1989). This phenomenon was attributed to a positive charge at the bottom of the active site, which may stabilise the negatively charged carboxyl group in head‐to‐tail orientation. Alternatively, an acidic pH may protonate the carboxyl group of the substrate leading to suppression of the negative charge. This hypothesis is supported by arginine residues at the bottom of the substrate channel of certain LOX, which require head‐to‐tail orientation of the substrate in the case of the lipid body 13*S‐*LOX from cucumber (Cs‐LBLOX) and the 13*S*‐LOX1 from soybean (GmLOX1; Hornung *et al*. 1999; Ruddat *et al*. 2004). In addition, histidines in the centre of the substrate channel of mammalian LOX may play a similar role (Newcomer & Brash 2015). Additionally, it was proposed that the protonation of the carboxyl group at acidic pH would allow its penetration into the non‐polar channel, which would otherwise not be possible because of the negative cost to bury a charged group. This hypothesis is also supported by the rather acidic pH optimum of LOX, which seem to bind their substrate in head‐to‐tail orientation (Butovich *et al*. 1998) and by the high p*K*a (pH 7–8) of PUFA in solution (Glickman & Klinman 1995; Kanicky & Shah 2002, 2003).

The regiospecific oxidation of PUFA is a suitable tool used so far by multicellular organisms in signalling pathways. Yet, apart from animals and flowering plants, LOX were also found in bryophytes (Anterola *et al*. 2009; Kanamoto *et al*. 2012; Senger *et al*. 2005), fungi (Brodhun *et al*. 2013; Wennman *et al*. 2015a,2015b) and prokaryotes (Andreou *et al*. 2008, 2010; Vance *et al*. 2004; Zheng *et al*. 2008). While a large body of evidence exists on the function of many LOX in vertebrates and flowering plants, knowledge on the function of LOX in eukaryotic algae is scarce (Andreou *et al*. 2009), although LOX activity was described for the green alga *Chlorella pyrenoidosa* more than 40 years ago (Bisakowski *et al*. 1995; Zimmerman & Vick 1973). Recently, the first sequences encoding putative LOX were detected in the transcriptomic data of two green microalgae, C*hlamydomonas reinhardtii* (Merchant *et al*. 2007) and *Lobosphaera incisa* (Siegler *et al*. 2017). This study aims to characterise, to the best of our knowledge, the first LOX (LiLOX) from a green microalga (*L. incisa*) to understand the basic mechanism LiLOX uses to achieve selective oxidation of PUFA, and to obtain first insights on the role of the first LOX in green microalgae.

## Material and methods

### Chemicals

Methanol and *n*‐hexane were purchased from ACORS (ThermoFisher Scientific, Waltham, MA, USA), diethyl ether and acetic acid from Carl Roth & Co (Karlsruhe, Germany), propan‐2‐ol from Fisher chemicals (ThermoFisher). All additional chemicals were obtained from Carl Roth. Restriction enzymes were all obtained from MBI Fermentas (ThermoFisher) and PUFA substrates as well as standards (HODE, HOTE and HETE) were purchased from Cayman Chemical (Ann Arbor, MI, USA).

### Phylogenetic analysis of LiLOX

Phylogeny of LOX was based on protein sequence homology. All accession numbers were obtained from GeneBank: *Arabidopsis thaliana* LOX1 (9*S*) (NP_175900.1), *A. thaliana* LOX2 (13*S*) (NP_566875.1), *A. thaliana* LOX3 (13*S*) (NP_564021.1), *A. thaliana* LOX4 (13*S*) (NP_177396.1), *A. thaliana* LOX5 (9*S*) (NP_188879.2), *A. thaliana* LOX6 (13*S*) (NP_176923.1), *Burkholderia thailandensis* (13*S*) (ABC36974), *C. reinhardtii* LOX (putative) (XP_001690882.1), *Cyanothece *sp. LOX1 (9*R*) (WP_012595715.1), *Cyanothece* sp. LOX2 (11*R*) (WP_012596348.1), *Fusarium oxysporum* LOX (13*S*) (EXK38530), *G. fructosovora* LOX (11*R*) (AAY98506.1), *G. graminis* Mn‐LOX (13*R*) (AAK81882.1), *Glycine max* LOX1 (13*S*) (NP_001236153.1), *G. max* LOX2 (9/13*S*) (NP_001237685.1), *G. max* LOX3 (9/13*S*) (CAA30016), *G. max* LOX4 (13*S*) (BAA03101), *Homo sapiens* LOX2 (15*S*) (AAB61706.1), *H. sapiens* LOX (5*S*) (NP_000689.1), *H. sapiens* LOX (12*R*) (NP_001130.1), *H. sapiens* LOX (12*S*) (NP_000688.2), *H. sapiens* LOX (15*S*) (NP_001131.3), *Magnaporthe salvinii* Mn‐LOX (9*S*) (CAD61974.1), *Nostoc punctiforme* LOX (9*R*) (WP_010994078.1), *N. punctiforme* LOX2 (13*S*) (WP_012407347.1), *Oryza sativa* LOX2 (13*S*) (NP_001067011.1), *O. sativa* LOX (13*S*) (EEC72668), *Pseudomonas aeruginosa* LOX (15*S*) (WP_023436288.1), *P. homomalla* LOX (8*R*) (AAC47743), *Physcomitrella patens* LOX1 (13*S*) (XP_001784705.1), *P. patens* LOX2 (13*S*) (ABF66648), *P. patens* LOX3 (13*S*) (XP_001785004.1), *P. patens* LOX4 (13*S*) (ABF66650.1), *P. patens* LOX5 (13*S*) (ABF66651.1), *P. patens* LOX6 (13*S*) (ABF66652.1), *P. patens* LOX7 (13*S*) (ABF66653.1), *Solanum tuberosum* LOX (9*S*) (NP_001275357.1), *S. tuberosum* LOX (13*S*) (NP_001274843.1), *S. tuberosum* LOX2 (13*S*) (NP_001275115.1). The multiple alignment as well as the phylogenetic tree were performed using the software tool Genious, MUSCLE alignment with default parameters.

### Algal growth conditions and cDNA synthesis

*Lobosphaera incisa* strain SAG 2468 was used for this study. The algal cells were grown at constant 25 °C with maximum volume of 300 ml BG11 medium inside glass columns. A constant light of 190 μmol·photons·m^−2^·s^−1^ was provided to the cells. Carbon dioxide (1%) was the only source of carbon available to the algae, provided through a constant flow also used to agitate the cells to homogeny in the medium (Siegler *et al*. 2017). Total RNA of *L. incisa* was obtained from 20 mg dry cells using TRIzol (ThermoFisher Scientific; Siegler *et al*. 2017). RevertAid RT (ThermoFisher Scientific) was used to obtain cDNA, using oligo(dt)18 as primers.

### Cloning

The RACE‐PCR was performed using the SMARTer^®^ RACE 5′/3′ Kit (Takara, Clontech, Heidelberg, Germany) according to the manufacturer's instructions. Inner primers: LiLOX Forward GGCATCGGCGCGTGAGGCAG, Reverse GACTACCCCTATGCAGCCGACG. LiLOX was amplified using the following primers, Forward GAATTCGACAGCGTGCTTCCCCATGGC, Reverse TTGCGGCCGCTTACATTGAGACGCTGGTTGGGAT. The PCR reaction was performed with Phusion High Fidelity DNA polymerase (ThermoFisher) and cloned into pET28a (Merck, Darmstadt, Germany) using pJET cloning vector (ThermoFisher). The integrity of cloned vectors was verified by DNA sequencing (GATC Biotech, Konstanz, Germany).

### Mutations

Mutations were performed with 18 cycles of PCR, with the followingprimersN702T/F703V: Forward ATCATCGAGGGCACGTCACTCCTGGCCGCTATGCC, Reverse GCGGCCAGGAGTGACGGTGCCCTCGATGATACCACC; R853L: Forward GGCTTCATGCCCAACCTGAGCCCGATGATCCGAAAGGC, Reverse TTTCGGATCATCGGGCTCAGGTTGGGCATGAAGCCTGA; R853M: Forward GGCTTCATGCCCAACATGAGCCCGATGATCCGAAAGGC, Reverse TTTCGGATCATCGGGCTCATGTTGGGCATGAAGCCTGA.

### Recombinant expression of LiLOX in *E. coli* and purification

A 5‐ml pre‐culture of *E. coli* BL21 Star (ThemoFisher) freshly transformed with LiLOX‐pET28a was added to 1 l Auto‐induction ZY medium (Studier 2005), cultivated at 37 °C for 2.5 h, then at 16 °C during 42.0 h, with constant 200 rpm shaking. After harvest, the cells were resuspended in the running buffer (0.02 m HEPES buffer, pH 8.4, 0.15 m NaCl), with addition of 25 mg lysozyme and 25 mg deoxy‐ribonuclease I. Lysis was performed after 1 h using Fluidizer (Microfluidics Corp., Westwood, MA, USA) three times at 60 psi. Lysate was centrifuged at 10 000 *g* for 10 min and the supernatant was applied on a 5 ml IMAC affinity chromatography HisTrap HP (GE Healthcare, Braunschweig, Germany) using ÄKTA prime. The column was rinsed with four column volumes (CV) of running buffer, then four CV with 5% Elution Buffer (0.02 m HEPES buffer, pH 8.4, 0.15 m NaCl, 0.5 m Imidazole) in order to wash off loosely bound proteins. LiLOX was finally eluted from the column using 50% Elution Buffer. The elution fractions showing LOX activity were combined and applied on size‐exclusion chromatography (SEC) HiLoad 26/60 Superdex 200 (GE Healthcare) using Gel filtration Buffer [0.02 m HEPES buffer, pH 8.4, 0.1 m NaCl, 2% glycerol (v/v)]. The purified protein was stored at −80 °C for up to a year.

### Kinetics

The LOX activity was measured *via* absorbance increase at 234 nm (molar extinction coefficient of conjugated diene system: ε = 2.5 × 10^4^ m ^−1^·cm^−1^) using a spectrophotometer CARY 100 Bio (Varian, Dramstadt, Germany) as described (Newie *et al*. 2017). Unless otherwise specified, all kinetic reactions were measured in 1 ml 20 mm Bis‐TRIS propane buffer at 30 °C (Newie *et al*. 2017). Reactions were started by adding 1 μg LiLOX. To determine optimum pH, LiLOX activity was measured with constant substrate concentration (100 μm) with varying pH from 5.5 to 9.5. To determine the kinetic parameters, constant pH 7.5 was applied while substrate concentration varied from 10 to 100 μm. The formation of conjugated triene was measured following absorbance at 271 nm.

### Fatty acid extraction

Prior to analysis, oxidised fatty acids were reduced to the corresponding hydroxides with Na borohydride for 5 min. Reduction reaction was stopped with addition of acetic acid (5%, v/v). Oxidation products were extracted with an equal volume of diethyl ether, strongly vortexed for 30 s and centrifuged at 1000 × *g* for 1 min. The hydrophobic phase was dried under a stream of nitrogen and solubilised in 200 μl methanol.

### Regio‐ and stereospecificity determined by HPLC

Oxidation products were analysed as described (Newie *et al*. 2017) on straight phase (SP) column Zorbax RX‐SIL; (150 × 2.1 mm, particle size 5 μm; Agilent, Waldbronn, Germany) *via* high pressure liquid chromatography (HPLC) with constant flow rate of 0.2 ml·min^−1^ solvent system containing *n*‐hexane/2‐propanol/trifluoroacetic acid (100:1:0.1, v/v/v) coupled to a diode array detector. After collection of the compounds of interest, stereoisomers were separated on chiral phase (CP) column CHIRALCEL OD‐H (150 × 2.1 mm, Agilent) with constant flow rate 0.1 ml·min^−1^. The solvent system ratio of *n*‐hexane/2‐propanol/trifluoroacetic acid differed according to the hydroxide to be analysed. The separation of stereoisomers was achieved with a solvent ratio 100:5:0.1 for all HODE and HOTE isomers; 100:2:0.1 (v/v/v) for 8‐HETE, 12‐HETE, 15‐HETE; and 100:1:0.1 (v/v/v) for 5‐HETE and 11‐HETE. All isomers were identified with authentic standards beside the stereoisomers 11*R*‐hydroxy hexadecatrienoic acid (HHTE)/11*S*‐HHTE and 11*R*‐hydroxy hexadecadienoic acid (HHDE)/11*S*‐HHDE, were tentatively assigned. Absorbance was constantly recorded at wavelength of 234 nm and 220 nm.

### Analysis of Di‐HETE

For analysis of Di‐HETE products, mono‐hydroxy fatty acids and di‐hydroxy fatty acids were separated on reverse phase HPLC (RP‐HPLC), using the column EC250/2 Nucleosil C_18_ (length 250 mm, inner diameter 4 mm, particle size 5 μm; Macherey‐Nagel, Düren, Germany). A mixture of methanol:water:acetic acid, with a ratio of 75:25:0.1 (v/v/v) for solvent A and ratio of 100:0:0.1 (v/v/v) for the solvent B, was applied. The separation was performed according to the following gradient: 100% Solvent A from 0 to 10 min at 0.18 ml·min^−1^. Gradient from 0% to 100% buffer B for 15 min. The flow rate increase to 0.36 ml·min^−1^ within 2 min, and the flow remained isocratic from 27 to 32 min, followed by a gradient from 0% to 100% solvent A within 5 min. From 37 to 40 min, the flow rate slowed to 0.18 ml·min^−1^ and the method was complete after 46 min.

## Results

### Cloning

From analysis of the complete genomic data of the green microalga *L. incisa*, along with protein‐coding gene prediction and annotations (Siegler *et al*. 2017), open reading frames (ORF) of two putative LOX, one 450 nucleotides shorter at the 5′‐end than the second, were identified. In order to determine the endogenous occurrence of those transcripts, a RACE‐PCR was performed on cDNA of *L. incisa*. The sequencing of both 5′‐ and 3′‐ends (File S1) revealed that most likely only the longer sequence of this transcript, corresponding to 994 amino acids, naturally occurs in *L. incisa*. Nevertheless, both putative transcripts were cloned in the expression vector pET28a for heterologous protein expression in *E. coli*. Although the shorter version of LiLOX (844 amino acid residues) was successfully purified, it showed no LOX activity (data not shown). On the other hand, enzymatic activity after a spectrophotometric assay confirmed LOX activity for the longer version of the protein (994 amino acid residues, Genbank accession no: MG948464). As it represents the only known LOX from *L. incisa*, this protein was named LiLOX.

### Alignment of LOX shows highest homology with chloroplast LOX

**Figure 2:**
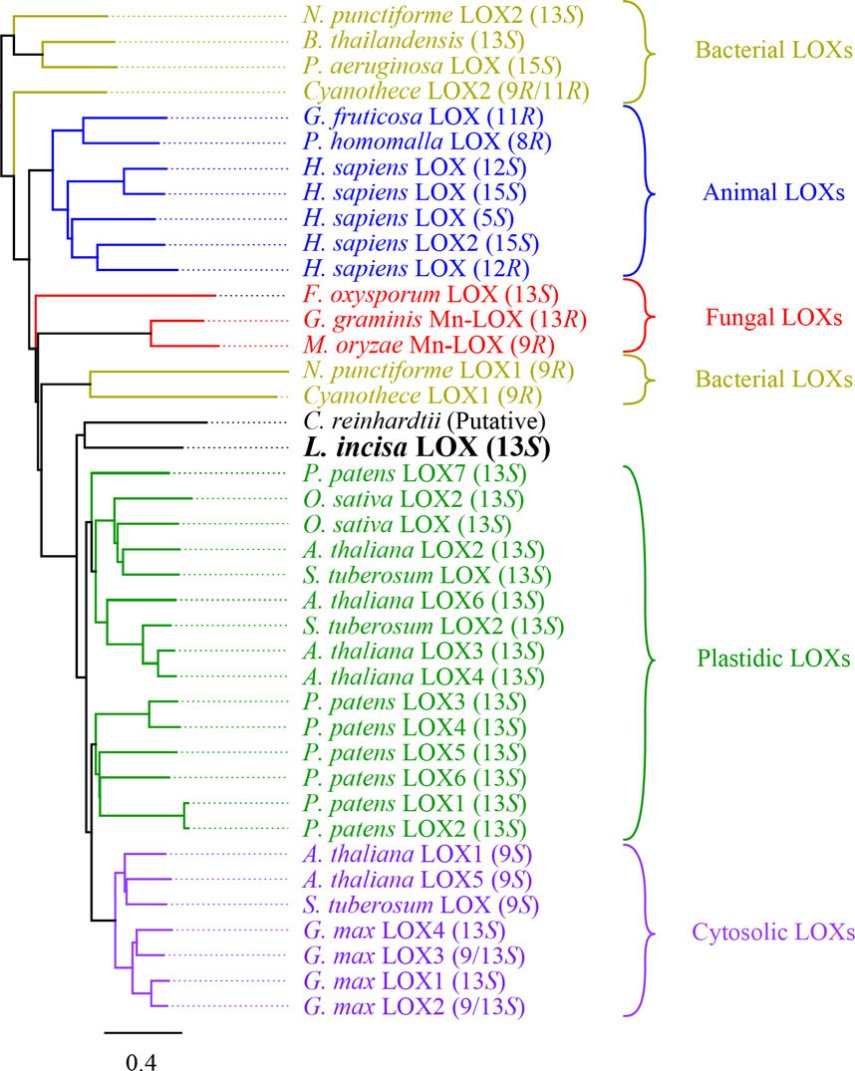
Phylogenetic three of LOX based on protein sequence homology. All accession numbers were obtained from GeneBank: *A. thaliana* LOX1 (9*S*) (NP_175900.1), *A. thaliana* LOX2 (13*S*) (NP_566875.1), *A. thaliana* LOX3 (13*S*) (NP_564021.1), *A. thaliana* LOX4 (13*S*) (NP_177396.1), *A. thaliana* LOX5 (9*S*) (NP_188879.2), *A. thaliana* LOX6 (13*S*) (NP_176923.1), *B. thailandensis* (13*S*) (ABC36974), *C. reinhardtii* LOX (putative) (XP_001690882.1), *Cyanothece* sp. LOX1 (9*R*) (WP_012595715.1), *Cyanothece* sp. LOX2 (11*R*) (WP_012596348.1), *F. oxysporum* LOX (13*S*) (EXK38530), *G. fructosovora* LOX (11*R*) (AAY98506.1), *G. graminis* Mn‐LOX (13*R*) (AAK81882.1), *G. max* LOX1 (13*S*) (NP_001236153.1), *G. max* LOX2 (9/13*S*) (NP_001237685.1), *G. max* LOX3 (9/13*S*) (CAA30016), *G. max* LOX4 (13*S*) (BAA03101), *H. sapiens* LOX2 (15*S*) (AAB61706.1), *H. sapiens* LOX (5*S*) (NP_000689.1), *H. sapiens* LOX (12*R*) (NP_001130.1), *H. sapiens* LOX (12*S*) (NP_000688.2), *H. sapiens* LOX (15*S*) (NP_001131.3), *M. salvinii* Mn‐LOX (9*S*) (CAD61974.1), *N. punctiforme* LOX (9*R*) (WP_010994078.1), *N. punctiforme* LOX2 (13*S*) (WP_012407347.1), *O. sativa* LOX2 (13*S*) (NP_001067011.1), *O. sativa* LOX (13*S*) (EEC72668), *P. aeruginosa* LOX (15*S*) (WP_023436288.1), *P. homomalla* LOX (8*R*) (AAC47743), *P. patens* LOX1 (13*S*) (XP_001784705.1), *P. patens* LOX2 (13*S*) (ABF66648), *P. patens* LOX3 (13*S*) (XP_001785004.1), *P. patens* LOX4 (13*S*) (ABF66650.1), *P. patens* LOX5 (13*S*) (ABF66651.1), *P. patens* LOX6 (13*S*) (ABF66652.1), *P. patens* LOX7 (13*S*) (ABF66653.1), *S. tuberosum* LOX (9*S*) (NP_001275357.1), *S. tuberosum* LOX (13*S*) (NP_001274843.1), *S. tuberosum* LOX2 (13*S*) (NP_001275115.1).

**Figure 3:**
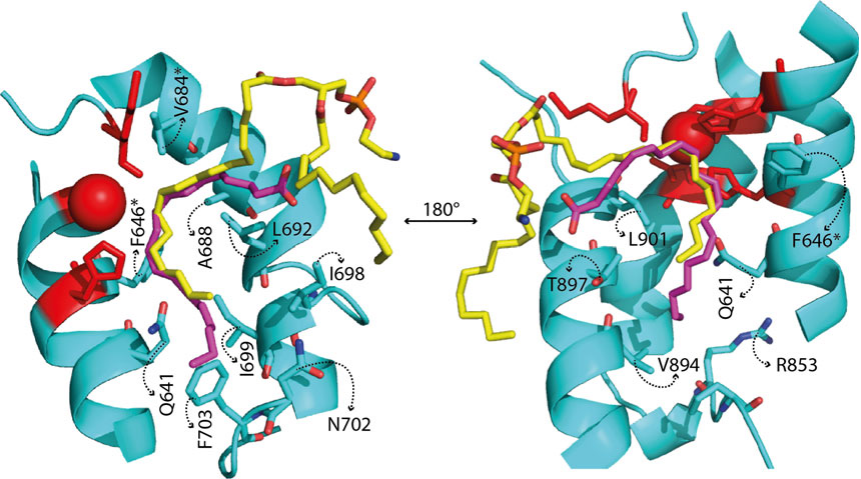
Conserved amino acids in the predicted structure of LiLOX (cyan). The modelled structure of LiLOX was superimposed with *P. homomalla* LOX (PhLOX, PDB accession no: 4QWT), and *P. aeruginosa* LOX (PaLOX, PDB accession no: 5IR5), the three structures were aligned to one another using the software PyMOL. The substrates co‐crystalised in the substrate channel of PaLOX and PhLOX are revealed (Magenta 20:4(n‐6) and yellow phosphatidylethanolamine, respectively). Iron binding residues are depicted in red.

Already characterised LOX used in a recently published phylogenetic tree were used as template, to which the protein sequence of LiLOX was added (Newie *et al*. 2016). LiLOX was found to have the highest homology with plastidial LOX from *Physcomitrella patens* (up to 32.6 % homology with PpLOX7), and *A. thaliana* (31.5% and 31.3% homology with AtLOX6 and AtLOX3, respectively). LiLOX is to the best of our knowledge the first green algal LOX to be characterised experimentally, but for comparison the protein sequence of a putative LOX from *C. reinhardtii* (Merchant *et al*. 2007) was added to the alignment. It was found to group together with LiLOX, possibly forming a clade specific for green microalgal LOX (Fig. 2). Since no structural data are available for plastidic LOX, the 3D structure of LiLOX was modelled using the software Phyre2 (Fig. 3). This structure prediction was superimposed with previously available LOX structures, confirming similarities in the active site region (File S2) and allowing us to derive the first hypotheses on the conformation of the substrate channel.

### The LiLOX shows highest activity with PUFA 18:3(n‐3)

**Figure 4:**
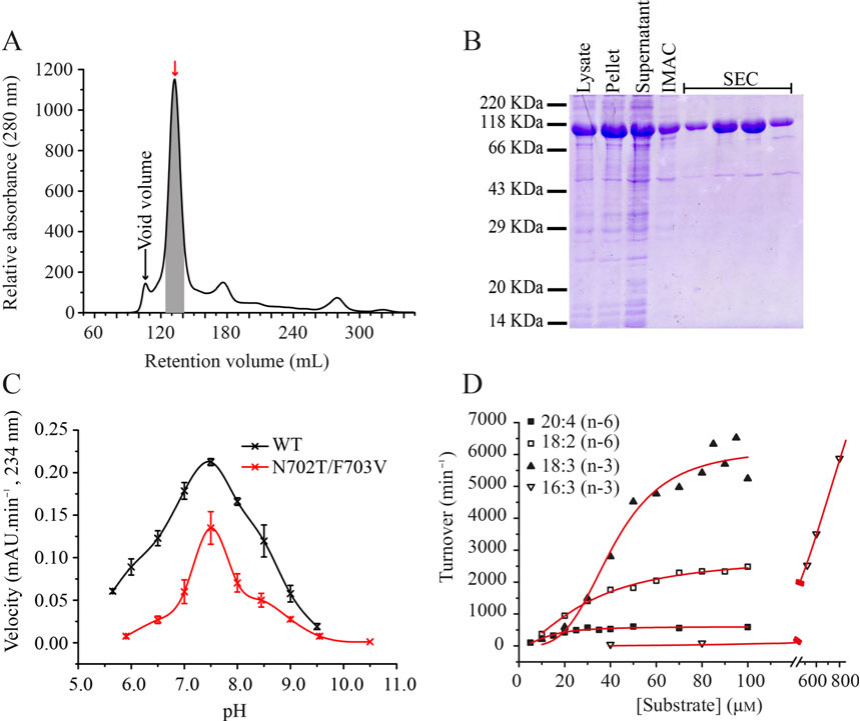
Purification and activity analysis of *L. incisa* LOX (LiLOX). LiLOX cloned without the N‐terminal putative signal peptide and fused to an N‐terminal His tag was heterologously expressed in *E. coli* BL21 star cells. After expression in auto‐induction medium, cells were disrupted by sonication. LiLOX was purified by Immobilized Metal Affinity Chromatography (IMAC) and size exclusion chromatography (SEC) performed on HiLoad 26/60 Superdex S200 SEC‐column. LOX activity was determined *via* spectrophotometer, measuring the increase of product with a conjugated double bond system at 234 nm. A: SEC in 20 mm HEPES buffer, pH 8.5, 100 mm NaCl and 2% glycerol. The chromatogram shows the absorption at 280 nm during elution, the grey area indicates the four fractions containing detectable LiLOX activity, also depicted with a red arrow. B: SDS‐PAGE, illustrating the proteins of the *E. coli* lysate, supernatant and pellet fractions, IMAC fraction as well as the four SEC fractions from Fig. 4A containing LiLOX. Protein purification performed at least five times giving comparable results. C: Spectrophotometric measurement of the pH optimum of LiLOX (black squares) and its N702T/F703V mutant (red dots). 20:4(n‐6) was used as substrate at a concentration of 100 μm. 20 mm Bis‐TRIS propane was used as buffer. 1 μg pure LiLOX wild type and 2 μg pure LiLOX N702T/F703V mutant were used to determine the initial slope of the LOX activity at a given pH. The reaction was detected as increase in absorbance at 234 nm. Error bars represent the SD for three reactions; all three reactions were performed with the same enzyme preparation. D: Kinetic plots for polyunsaturated fatty acid (PUFA) conversion by LiLOX. The PUFA 20:4(n‐6), 18:2(n‐6), 18:3(n‐3) and 16:3(n‐3) were used as substrate at a concentration from 10 to 100 μm (800 μm for 16:3(n‐6)). 20 mm Bis‐TRIS propane was used as buffer pH 7.5, 1 μg pure LiLOX was used to determine the initial slope of LiLOX activity at a given substrate concentration. The reaction was detected as increase in absorbance at 234 nm at 30 °C. For the PUFA 18:3(n‐3), each point represents the mean value of three reactions. Each reaction was performed with different enzyme preparations. For the PUFAs 18:2(n‐6) and 20:4(n‐6), each point represents the mean value of two replicates.

As the protein identity suggested that LiLOX shares highest identity with plastidial LOX, an *in silico* prediction was performed with ChloroP1.1 (Emanuelsson *et al*. 1999) to determine the presence and length of a putative signal peptide. Indeed, this algorithm predicted a plastidial localisation of LiLOX. For protein expression and characterisation of LiLOX, the cDNA was cloned into the pET28a expression vector without the putative signal peptide corresponding to 62 amino acids at the N‐terminus, following the prediction of ChloroP1.1 (Emanuelsson *et al*. 1999). LiLOX was then heterologously expressed in *E. coli*, before purification by affinity chromatography and size exclusion chromatography (Fig. 4A). To ensure the purity of LiLOX during the purification process, samples were stored after each step for SDS electrophoresis (Fig. 4B).

After purification of LiLOX, its activity was measured with a spectrophotometer by recording the increasing absorbance at 234 nm after addition of 1 μg pure enzyme. Since *L. incisa* is known to accumulate high amounts of 20:4(n‐6) under nitrogen starvation (Bigogno *et al*. 2002; Khozin‐Goldberg *et al*. 2002), this PUFA was used to determine the pH optimum of LiLOX. Using spectrophotometric assay, LiLOX was found to have highest activity at pH 7.5 in BisTris propane buffer (Fig. 4C). At this pH, the kinetic parameters were determined with three different PUFA, 18:2(n‐6), 18:3(n‐3), 20:4(n‐6), with substrate concentrations varying from 10 to 100 μm to avoid micelle formation. Since the obtained data did not fit the Michaelis‐Menten equation, the Hill equation was used instead for all three substrates (Fig. 4D). LiLOX was found to have the highest *k*_cat_ with 18:3(n‐3), as well as the highest *k*_cat_/*K*_M_ ratio (Table 1). Notably, LiLOX was found to also be active towards 16:3(n‐3), yet since the data fitted neither the Michaelis‐Menten equation nor the Hill equation, kinetic parameters for this substrate could not be determined.

**Table 1:** Kinetic parameters of LiLOX with PUFA

substrate	*k*_cat_ (min^−1^)	SD	*K*_M_ (μm)	SD	*k*_cat_/*K*_M_ (μm ^−1^·min^−1^)	hill coefficient	SD
18:2(n‐6)	2834.92	157.67	31.16	2.78	90.98	1.58	0.16
18:3(n‐3)	7432.84	574.41	55.55	4.10	133.80	2.61	0.28
20:4(n‐6)	606.06	27.25	12.64	1.1	47.95	1.96	0.31

Varying substrate concentrations were used from 10 to 100 μm, and 20 mm BisTRIS propane pH 7.5 was used as buffer. The reaction was detected as increase in absorbance at 234 nm after addition of 1 μg purified LiLOX at 30 °C. All kinetic parameters were determined using the software OriginPro8.5. Data derived from the analysis shown in Fig. 4D.

### The LiLOX oxidises all PUFA at carbon n‐6 with *S* configuration

In addition to 18:2(n‐6), 18:3(n‐3) and 20:4(n‐6), the oxidation products from two more substrates were analysed: 16:3(n‐3) and 18:3(n‐6). In order to determine the oxidation specificity of LiLOX, all fatty acid hydroperoxides formed were reduced into hydroxy fatty acids, extracted and sequentially analysed by SP‐HPLC and CP‐HPLC. All substrates were found to be oxidised by LiLOX in a regio‐ and stereospecific manner, resulting in 11*S*‐hydroxy hexadecatrienoic acid (HHTE), 13*S*‐HODE, 13*S*‐α‐hydroxy octadecatrienoic acid (HOTE), 13*S*‐γ‐HOTE and 15*S*‐hydroxy eicosatetraenoic acid (HETE; summarised in Table 2, File S3). These results highly suggest a so‐called tail‐first orientation of the substrate in the active site of LiLOX. Indeed, from the methyl end, the oxidation by LOX primarily happens in the position n‐6, regardless of the acyl chain, suggesting that the hydrogen abstraction occurs on the carbon n‐8.

**Table 2:** Oxidation products from LiLOX with different PUFA

substrate	positional specificity (chiral conformation)					
16:3(n‐3)	11‐HHTE 97.9 (100% *S*)	7‐HHTE 2.1	14‐HHTE n.d.	10‐HHTE n.d.		
18:2(n‐6)	13‐HODE 98.5 (97.9% *S*)	9‐HODE 1.5				
18:3(n‐3)	13‐HOTE 96.4% (100% *S*)	12‐HOTE 3.6	16‐HOTE n.d.	9‐HOTE n.d.		
18:3(n‐6)	13‐HOTE 98.4% (97.8% *S*)	10‐HOTE 0.6	9‐HOTE 0.9	6‐HOTE n.d.		
20:4(n‐6)	15‐HETE 99.4% (97.8% *S*)	12‐HETE 0.2	11‐HETE 0.4	8‐HETE n.d.	9‐HETE n.d.	5‐HETE n.d.

LiLOX shows strict regio‐ and stereospecificity of oxidation in position n‐6 with *S* configuration. Data represent the mean of three measurements. Each measurement was performed with a different enzyme preparation. n.d., not detected.

### The mutants N702T and F703V show oxidation variability

**Figure 5:**
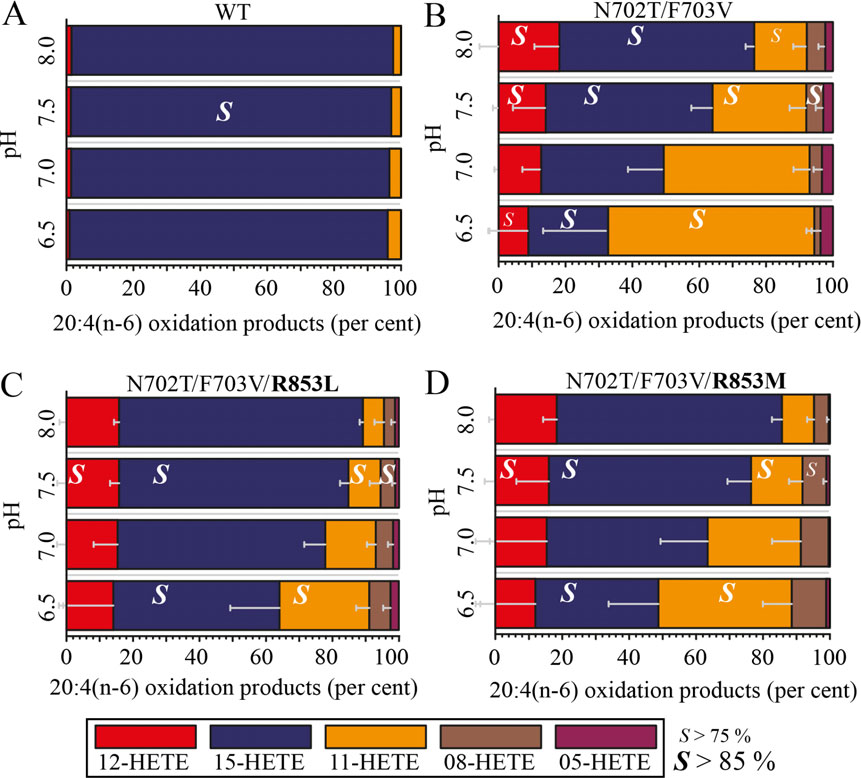
Effect of the pH and mutations on the oxidation products of LiLOX with 20:4(n‐6). Reaction products were identified by SP‐HPLC. 20 mm Bis‐TRIS propane was used as buffer for all reactions with 100 μm PUFA 20:4(n‐6). Each bar represent the amount of an oxidation product relative to the others (%). The error bars represent SD for three independent experiments, performed with different enzyme preparations. The ratio of the two stereoisomers of each compound was determined by CP‐HPLC and the result is given in each box, representative of one measurement. A: LiLOX WT; B: LiLOX N702T/F703V; C: LiLOX N702T/F703V/R853L; D: LiLOX N702T/F703VR853M.

**Figure 6:**
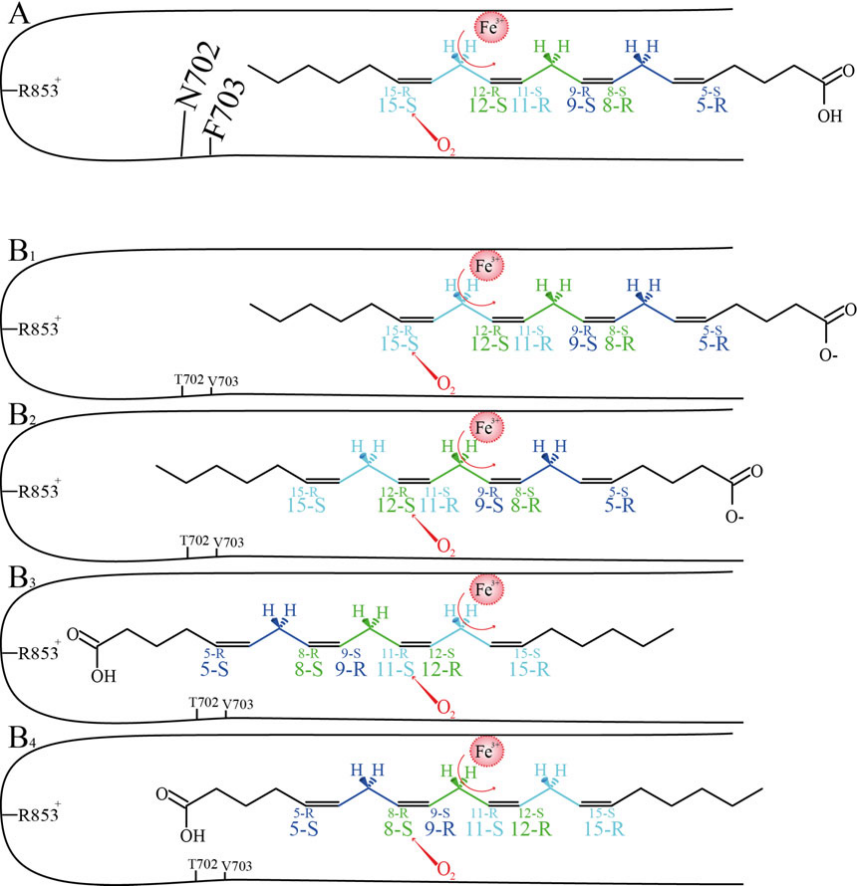
Models to explain the oxidation products formed in the substrate channel of LiLOX and its mutants, assuming that the direction of the molecular oxygen (red) is not affected by the mutations. The colours highlight the three *bis*‐allylic carbon groups present in this PUFA. Four oxidation products are possible after one hydrogen has been abstracted on a *bis‐*allylic carbon atom, depicted in the corresponding colour. The representations of two putative stereoisomers are differentiated by the size of the font: small (behind the plane) or big (above the plane). A: 20:4(n‐6) oxidation products by wild type LiLOX. B1–4: Four different 20:4(n‐6) oxidation products by the LiLOX N702T/F703V mutant.

In order to confirm the hypothesis of a tail‐first substrate orientation, the regiospecificity of LiLOX was investigated after point mutations of conserved amino acid residues aligning at the bottom of the binding pocket within the active site of the enzyme. After protein alignments with LOX from different organisms, the corresponding determinants were found to be conserved, as shown for the second and third amino acid motif in File S4 and consistent with other 13*S*‐LOX. In order to increase the depth of the LOX substrate channel, the two respective residues N702 and F703 in the second motif were mutated into smaller residues in order to challenge this hypothesis (Fig. 3). As new amino acid residues, threonine and valine were chosen because these residues are highly conserved in plant LOX having a larger binding pocket (Hornung *et al*. 1999). Since the wild‐type enzyme only produces 15*S*‐HETE from the PUFA 20:4(n‐6) (Fig. 5A), the mutation N702T/F703V is expected to increase the depth of the substrate channel and therefore allow a substrate frame‐shift and the oxidation products should then be 15*S*‐HETE, 12*S*‐HETE or 9*S*‐HETE (Fig. 6). Instead, the major species observed were found to be 15*S*‐HETE and 11*S‐*HETE (Fig. 5B). The very presence of 11*S*‐HETE in significant amounts, however, highly suggests an inversion of the substrate in the active site of LiLOX, as this is not possible with a tail‐first insertion (Mortimer *et al*. 2006). Currently, two models attempt to explain the possibility of burying a polar carboxyl group in the non‐polar substrate channel. First, a polar amino acid at the bottom of the substrate channel stabilises the carboxyl group with ion‐to‐ion, ion‐to‐dipole or dipole‐to‐dipole interactions. In the case of LiLOX, this could be explained by the presence of R853, homologous to R758 and R707 in Cs‐LBLOX and GmLOX1, respectively (Hornung *et al*. 1999; Ruddat *et al*. 2004), which may become accessible to the carboxy group of the substrate after N702T/F703V mutation. Second, the pH may play a role in protonating the fatty acid substrate, rendering it non‐charged, thus less polar and more likely to penetrate the non‐polar substrate cavity of LiLOX (Fig. 6). In order to test the two theories, both were challenged by measuring the product specificity at different pH values.

### The pH affects regiospecificity of the LiLOX N702T/F703V mutant

Before testing the influence of pH on the product specificity of LiLOX wild type and mutant N702T/F703V, it was shown that the pH optimum with regard to enzyme activity was unaffected by the mutation (Fig. 4). Nevertheless, variations in the pH did affect the shares of oxidation products from the mutant, especially the ratio of 15*S*‐HETE to 11*S*‐HETE, which is high at basic pH and low at acidic pH (Fig. 5B, File S5). Such an effect was not observed on LiLOX WT (Fig. 5A). This result seems to be concordant with the protonation of the fatty acid theory, more likely to perform head‐to‐tail orientation at acidic pH.

In order to demonstrate the effect of the putative positive charge R853 on the head‐to‐tail orientation (Fig. 3), this residue was mutated into a non‐polar leucine and a non‐charged methionine in the LiLOX N702T/F703V mutant. As a result, 11*S*‐HETE was still produced but in lesser amount by LiLOX N702T/F703V/R853M and even less by N702T/F703V/R853L (Fig. 5C and D, File S5). Hence, despite the removal of the only basic residue putatively at the bottom of the substrate channel, LiLOX was still found to be able to produce 11*S‐*HPETE from 20:4(n‐6), although in smaller quantities. This suggests that, if a basic residue at the bottom of the substrate channel might increase chances for LOX reaction, the substrate is still able to enter the LOX channel by head‐to‐tail orientation, spontaneously, despite the absence of any accessible basic residue. Furthermore, the pH was still found to affect, but less, the 15*S*‐HETE/11*S*‐HETE ratio. This supports the fact that revealing the basic R853 residue increased the chances for 11*S‐*HETE formation, although not alone responsible for the head‐to‐tail orientation.

### Formation of Di‐HETE

**Figure 7:**
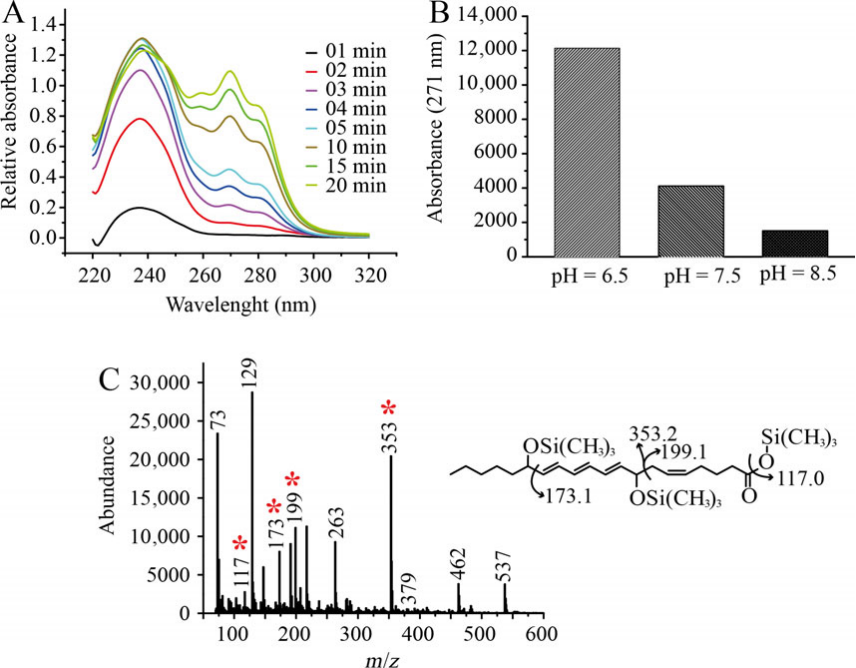
Di‐HETE formation by the *L. incisa* LOX mutant N702T/F703V with the PUFA 20:4(n‐6). 20 mm BisTRIS propane was used as buffer, and 100 μm 20:4(n‐6) was incubated for 1 h with 1 μg pure LiLOX N702T/F703V mutant. Results were obtained from a single experiment. A: Kinetic measurement by spectrophotometer, recording the reaction from 220 to 330 nm, from 0 to 20 min. The baseline level is established after addition of LiLOX. B: Abundance of Di‐HETE at different pHs, represented by integrated peaks from the RP‐HPLC. C: Fragmentation of the expected Di‐HETE by GC‐MS/MS. Data shown represent a single experiment.

Oxidation of 20:4(n‐6) by N702T/F703V revealed a two‐step reaction (Fig. 7A). The formation of single oxidised products with a maximum absorbance at 234 nm, reached a maximum after 10 min reaction. From 10 min onwards, the single oxidised products were converted into Di‐HETE, as revealed by the presence of a conjugated triene absorbing light with a characteristic maximum at 271 nm. The identity of 8,15‐Di‐HETE was confirmed by mass spectrometry (Fig. 7C). This suggests two different oxidations with two different orientations of the substrate: one head‐first leading to an oxidation on the 8th carbon (n‐13), and one tail‐first leading to oxidation on position C15 (n‐6). After incubation of the mutant with 20:4(n‐6) at different pH, it was found that this Di‐HETE was mostly produced at acidic pH (Fig. 7B), confirming the high impact of pH on head‐to‐tail substrate orientation. Such Di‐HETE formation was only detected with the N702T/F703V form of LiLOX, never with the wild‐type enzyme.

## Discussion

This study reports, to the best of our knowledge, the first cloning of a cDNA and complete characterisation of a LOX from green microalgae. The phylogenetic tree analysis of characterised LOX (Fig. 2) suggests that LiLOX and plastid LOX from flowering plants and bryophytes might share a common ancestor. This is in agreement with the relationship of these two clades (Yoon *et al*. 2004). The endogenous subcellular localisation in the chloroplast would be consistent with its predicted plastidial signal peptide, its pH optimum at pH 7.5 (Robinson 1985; Tikhonov 2012) and its strict 13*S* oxidation of C18 PUFAs (Liavonchanka & Feussner 2006).

The fact that LiLOX oxidises all PUFA 16:3(n‐3), 18:2(n‐6), 18:3(n‐6) and 20:4(n‐6) with a consistent oxidation on the carbon n‐6, suggests that PUFA enter the substrate channel by their methyl group first (tail‐first).

### The mutants N702T/F703V allow head‐to‐tail orientation of 20:4(n‐6)

After mutating the residues N702 and F703 into smaller residues, 15*S*‐HPETE remained the major compound formed at pH 7.5. Yet in addition, two different mechanisms seem to arise. First, a substrate frame‐shift due to a deeper channel, previously observed by Sloane *et al*. (1991), could explain the formation of 12*S*‐HPETE. Second, an inverse orientation of the substrate (head‐to‐tail), previously described in some plant LOX (Feussner & Kühn 1995; Regdel *et al*. 1994) seems to be necessary to explain the formation of 11*S‐*HPETE and 8*S*‐HPETE. The head‐to‐tail orientation also seems necessary for the formation 8*S*,15*S*‐DiHETE, already reported for GmLOX1 (Coffa *et al*. 2005a), an enzyme described as accepting a double insertion of the substrate, tail‐first and head‐to‐tail. However, in contrast to GmLOX1, for LiLOX the formation of the first oxidation product is still detectable while the formation of the double oxidation product has already started (Fig. 7). This implies that the second oxygenation is occurring at a similar rate to the primary oxygenation.

The feasibility of a PUFA entering the non‐polar substrate channel by its carboxyl group first (head‐to‐tail) has been subject to much debate (Browner *et al*. 1998; Prigge *et al*. 1998), yet the theory became accepted with compelling evidence supporting it (Coffa & Brash 2004; Coffa *et al*. 2005a,2005b; Kühn 2000). Moreover, head‐to‐tail orientation would allow the formation of all observed oxidation products with a consistent dioxygen attack directed towards the bottom of the substrate channel (Fig. 6).

This further suggests that the N702T/F703V mutation affected the head‐to‐tail orientation. This seems to be in accordance with the position of the two mutated amino acid residues in this model, assumed to widen the bottom of the substrate channel in LiLOX, therefore affecting a shift in substrate penetration, a different substrate orientation or both. Although the other hypotheses appear far less likely, an influence of the mutation on dioxygen shielding, a putative dioxygen channel, radical localisation or radical trapping cannot be completely excluded without any structural information on LiLOX at the amino acid side chain level.

Assuming a head‐to‐tail orientation of the substrate, the preference of N702T/F703V for 11*S*‐HPETE formation can be explained by two factors: (i) since the mutation N702T/F703V is expected to widen the bottom of the substrate channel, this may favour the substrate frame‐shift deeper into the channel; (ii) all 8*S*‐HPETE formed by this enzyme might be further oxidised into 8,15‐DiHETE (Figs 6 and 7). However, all 11*S*‐HPETE produced cannot be oxidised further because only the *bis*‐allylic hydrogens at C‐7 and C‐10 can now be abstracted. Moreover, products that derive from this reaction have so far not been observed with LiLOX.

### Acidic pH favours head‐to‐tail orientation of the substrate

Consistent with previous findings (Gardner 1989; Hornung *et al*. 2008; Walther *et al*. 2009), all products formed requiring a head‐to‐tail orientation are preferentially formed at acidic pH. The ratio of 15*S*‐HETE/11*S*‐HETE especially, is ten times higher at pH 6.5 than at pH 8. As previously proposed (Coffa *et al*. 2005b), this phenomenon can be explained by the protonation of the PUFA in the acidic condition, decreasing the negative cost of head‐to‐tail orientation. This hypothesis is consistent with the present data, and the p*K*_a_ of a free fatty acid between pH 7 and 8 (Glickman & Klinman 1995; Kanicky & Shah 2002, 2003).

It must be noted that the formation of 8,15‐DiHETE can influence the observed share of the single oxidised products. Indeed, either or both 8*S*‐HPETE and 15*S*‐HPETE formed by N702T/F703V can be further oxidised into 8,15‐DiHETE, as described above. Hence, the measured shares of 8*S*‐HPETE and 15*S*‐HPETE most likely do not reflect the exact share of these compounds as produced by the enzyme. Nevertheless, the formation of 8,15‐DiHETE, which requires head‐to‐tail orientation, is mostly detected at acidic pH, supporting further the hypothesis that protonation of the PUFA favours head‐to‐tail orientation. Moreover, since the formation of Di‐HETE requires two different alignments of the substrate in the active site, one in opposite orientation, their syntheses do not influence the overall ratio tail‐first/head‐to‐tail observed in the single oxidised products.

### The residue R853 favours head‐to‐tail orientation

Additionally to protonation of the PUFA, the residue R853 being highly conserved in plant LOX, R707 in GmLOX1 (Gardner 1989), R747 in Cs‐LBLOX (Hornung *et al*. 1999), is suspected to favour the head‐to‐tail orientation by stabilising the carboxyl group inside the substrate channel during oxidation. Both mutants lacking the basic residue R853, N702T/F703V/R853M and N702T/F703V/R853L, showed decreasing shares of 11*S*‐HPETE products, confirming that the residue R853 favours the chances for oxidation during head‐to‐tail orientation. It is not clear why this trend is found to be less pronounced in the case of N702T/F703V/R853M, but it may be explained by an uncommon hydrogen bond between the two pairs of electrons on the sulphur atom and the carboxyl group from the substrate. However, since the ratio 15*S*‐HETE/11*S*‐HETE remained affected by the pH despite the lack of R853, the protonation of the substrate appears the principal reason for the head‐to‐tail orientation and pH dependency. It is therefore proposed that the head‐to‐tail orientation can occur spontaneously in the substrate channel, where an available only polar or basic residue can help to stabilise the substrate and improve chances for hydrogen abstraction.

### Conclusion

The LiLOX may represent a good model to study plastic LOX because it is stable after heterologous expression in *E. coli* and highly active *in vitro*. Moreover, as the first fully characterised LOX from green microalgae, it opens the possibility to study endogenous LOX pathways in these organisms. Considering the numerous LOX pathways in embryophytes and mammals and knowing the current high focus on lipid pathways from microalgae, this promising possibility should not be overlooked.

## Conflict of Interest

The authors declare that they have no conflict of interest.

## Supporting information


**File S1.** Shorter and longer versions of the putative LOX from *L. incisa*. The shorter version is shown in black. The longer version corresponds to the whole sequence, in red and black.

**File S2.** Identification of regioisomers and stereoisomers of LiLOX products. The reaction mixture contained 1 µg of purified LiLOX in 20 mm Bis‐TRIS propane buffer pH 7.5 and 100 µm of respective PUFA. Reactions took place for 1 h at room temperature with accessible oxygen. Products were reduced by sodium borohydride for 10 min at room temperature and extracted with diethylether. A–E: SPHPLC chromatogram of LiLOX products derived from the following PUFA acid substrates: A: 18:2(n‐6); B: 18:3(n‐6); C: 18:3(n‐3); D: 20:4(n‐6); E: 16:3(n‐3). Results shown are representative for three independent experiments. Each experiment was performed with different enzyme preparations. For each main product, the respective chromatogram of the Chiral Phase CP‐HPLC is represented in the corresponding box, representative of one measurement. All isomers were identified with authentic standards besides the Stereoisomers 11*R*‐HHTE/11*S*‐HHTE and 11*R*‐HHDE/11*S*‐HHDE which were tentatively assigned. All LiLOX products were detected at 234 nm.

**File S3.** Comparison of 20:4(n‐6) oxidation products of LiLOX N702T/F703V mutant at different pH. A–D: Regioisomers separated by SP‐HPLC in n‐hexane:isopropanol:TFA 100:1:0.1 (v/v/v). 20 mm Bis‐TRIS propane was used as buffer with 100 µm of 20:4(n‐6). Reaction happened at pH A: 8.0. B: 7.5. C: 7.0. D: 6.5. Chromatograms of CP‐HPLC in n‐hexane:isopropanol:TFA 100:2:0.1 (v/v/v) is given for all 4 HETEs obtained after reaction at pH 7.5 in each corresponding box.

**File S4.** Alignment of five conserved sequence motifs of LOXs. Residue numbers are those from the LiLOX sequence. The five amino acid residues responsible for iron binding are framed and located in the first, fourth and fifth motif. In addition, the second motif shows the arginine residue at position 689 that may interact with carboxylate residue from the fatty acid substrate and the third motif shows two residues that are located at the bottom of the substrate‐binding pocket (702 and 703). For every given residue, identity represents its homology between all 13S‐LOXs from Fig. 7 used in this alignment (Green = 100% < Gold < 50% ≤ Red). Allignment obtained from the software Geneious, Muscle allignment with default parameters. LOXs accession numbers are as indicated in Fig. 2.

**File S5.** Summary of the shares from regio‐ and stereoisomers, after oxidation by LiLOX and LiLOX mutants with PUFA 20:4(n‐6). All regioisomers from 20:4(n‐6) were separated by SP‐HPLC and integrated. Data represent the average of the percentage of each regioisomers, from three independent experiments, performed with different enzyme preparations. The regioisomers present in sufficient amount were collected and analyzed by CP‐HPLC to separate both stereoisomers. The share of *S* isomers are presented in percentage. Rac: racemic.
