# Supplementary material for: The green microalga Lobosphaera incisa harbours an arachidonate 15S‐lipoxygenase
Source: Plant Biol (Stuttg). 2018 Oct 24;21(Suppl Suppl 1):131–42. doi: 10.1111/plb.12920 (PMC6587457; doi:10.1111/plb.12920)
Supplement: Supplementary file 1 — File S1. Shorter and longer versions of the putative LOX from L. incisa. The shorter version is shown in black. The longer version corresponds to the whole sequence, in red and black. [file PLB-21-131-s001.pdf]

MELGLGARQAVLQPAAASRAPLGLLKAGHRAQQRRLPSVPSVSASASVTQGIPQTSFLQKARDSVLPHGAP
FTPTSKDSVQWTTTIYTTQLLRPEIHDLGFVEVLLASEDKEAVSERMTLKGFSRKPWTNLDGVPKWGAIFT
GTLMLPAAMAKPAVMFININHPPEGVQYFFANKIALDGPPGKTAHVDFVINSHVDSGPDAPRPFFTAQAYL
PHAPMPDYLAELREHELAILRGTAAAAKHERKGSERIYDYDVYNDLGTPTRSRPALGGDTLKYPRRLRTGR
RVVNGTEVAAKGKDWLPPDERFDDRKQENFDSRTLLATLPALSANLLAALVPAGLQKLLRTPGSEFMSISD
IEQLYSGNAAEDNEDNVLTNLVAPAAAPLAAILGLLTQNSAQGNTPQKVFEQLSDFNKEVVNGALDALVVS
LDDLGQSQRAAILHALSLTEKDFQEYAQAGTGRHEVHPVHANFVNLLQEVLHRLLHFSTPAVIREGREGAW
TTDEEWGREQLAGQNACMIHAIWNDAPIKDLPADSAITEAVLQGHLEGHSISQLLSGDKPRLFLIDYMKGF
KDYAEKIAAAHPGNVMYAGRAVLYLRTDGELVPVAIELQAPRRKLEAFTSADSPTIWLLAKCIFSSIDAGY
HQLISHFVRAHACTEPYIIATRRQLSVMHPVFKLLITHCRFTLNVNSNARQQLINAGGIIEGNFTPGRYAM
ELSSVVYGLTWTFDSQALPHDLVNRGVARREKDGTLKLLMADYPYAADGLLVWDAFVEWFDSYLRLYYDDE
VDGKRVTDDPEITAWWTEIQEKGHPDIKEGWPQLQTIADLTQILTTIAWIASAHHAAINYGQYDYSGFMPN
RSPMIRKAMPPKESDDFKTLAAQDAETAILPFLASPLQATQVMATLGLLSTHSENEEYINDLEHPYLMVGT
EAYNKYKEFLSRLKQAEDTIKKRNADSQGHVIRAGPDAIHYRLMNPSSITENGDVQRQGVTMSGIPTSVSM

Additional file 1. Shorter and Longer versions of the putative lipoxygenase from *L. incisa*. The shorter version is shown in black. The longer version corresponds to the whole sequence, in red and black.
