# Supplementary material for: The green microalga Lobosphaera incisa harbours an arachidonate 15S‐lipoxygenase
Source: Plant Biol (Stuttg). 2018 Oct 24;21(Suppl Suppl 1):131–42. doi: 10.1111/plb.12920 (PMC6587457; doi:10.1111/plb.12920)
Supplement: Supplementary file 2 — File S2. Identification of regioisomers and stereoisomers of LiLOX products. The reaction mixture contained 1 µg of purified LiLOX in 20 mm Bis‐TRIS propane buffer pH 7.5 and 100 µm of respective PUFA. Reactions took place for 1 h at room temperature with accessible oxygen. Products were reduced by sodium borohydride for 10 min at room temperature and extracted with diethylether. A–E: SPHPLC chromatogram of LiLOX products derived from the following PUFA acid substrates: A: 18:2(n‐6); B: 18:3(n‐6); C: 18:3(n‐3); D: 20:4(n‐6); E: 16:3(n‐3). Results shown are representative for three independent experiments. Each experiment was performed with different enzyme preparations. For each main product, the respective chromatogram of the Chiral Phase CP‐HPLC is represented in the corresponding box, representative of one measurement. All isomers were identified with authentic standards besides the Stereoisomers 11R‐HHTE/11S‐HHTE and 11R‐HHDE/11S‐HHDE which were tentatively assigned. All LiLOX products were detected at 234 nm. [file PLB-21-131-s002.pdf]

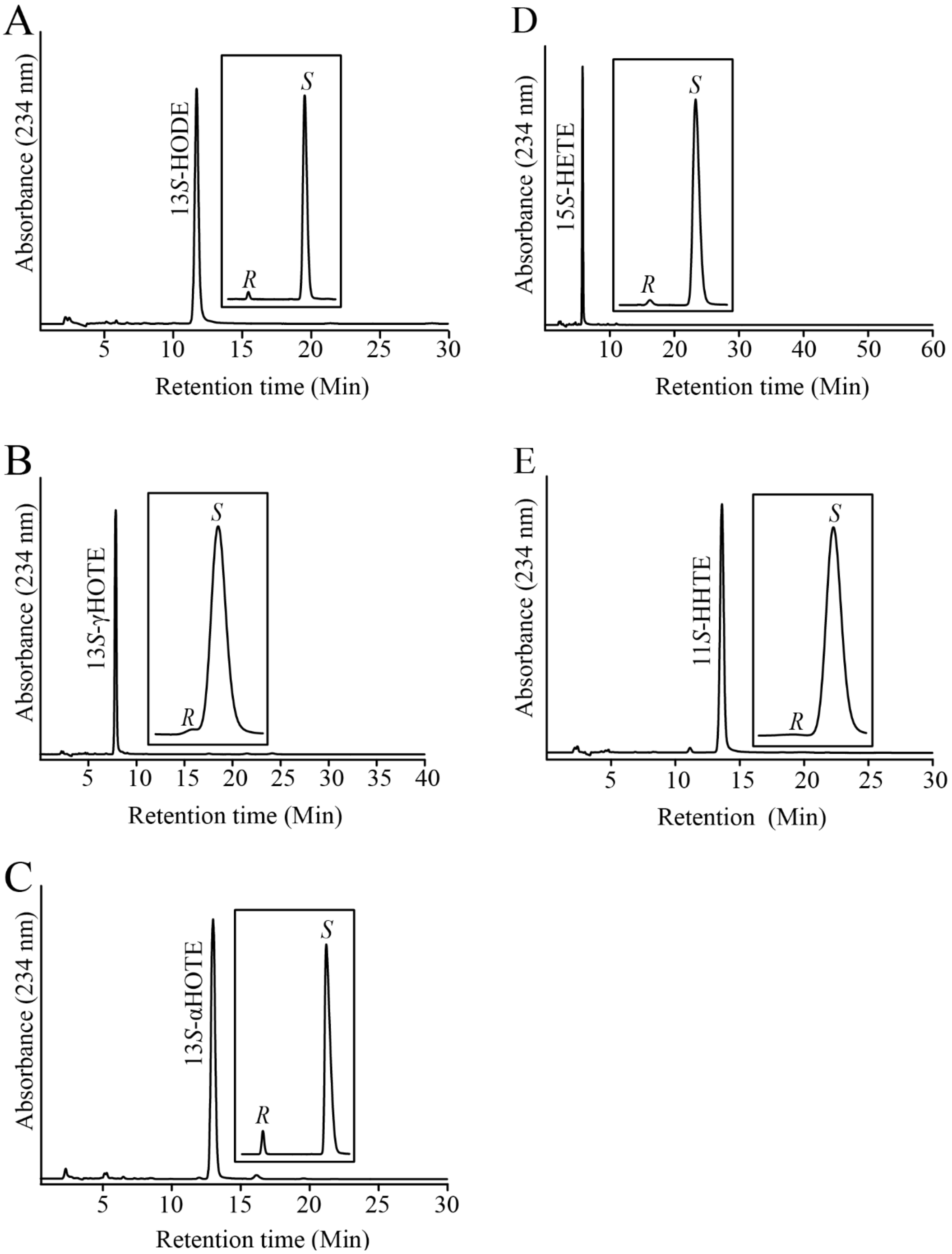

Additional file 2 Identification of regioisomers and stereoisomers of LiLOX products. The reaction mixture contained 1 µg of purified LiLOX in 20 mM Bis-TRIS propane buffer pH 7.5 and 100 µM of respective PUFA. Reactions took place for 1 h at room temperature with accessible oxygen. Products were reduced by sodium borohydride for 10 minutes at room temperature and extracted with diethylether. **A-E.** SPHPLC chromatogram of LiLOX products derived from the following PUFA acid substrates: **A.** 18:2(n-6); **B.** 18:3(n-6); **C.** 18:3(n-3); **D.** 20:4(n-6); **E.** 16:3(n-3). Results shown are representative for three independent experiments. Each experiments was performed with different enzyme preparations. For each main product, the respective chromatogram of the Chiral Phase CP-HPLC is represented in the corresponding box, representative of one measurement. All isomers were identified with authentic standards besides the Stereoisomers 11*R*-HHTE/11*S*-HHTE and 11*R*-HHDE/11*S*-HHDE which were tentatively asigned. All LiLOX products were detected at 234 nm.
