# Supplementary material for: The green microalga Lobosphaera incisa harbours an arachidonate 15S‐lipoxygenase
Source: Plant Biol (Stuttg). 2018 Oct 24;21(Suppl Suppl 1):131–42. doi: 10.1111/plb.12920 (PMC6587457; doi:10.1111/plb.12920)
Supplement: Supplementary file 3 — File S3. Comparison of 20:4(n‐6) oxidation products of LiLOX N702T/F703V mutant at different pH. A–D: Regioisomers separated by SP‐HPLC in n‐hexane:isopropanol:TFA 100:1:0.1 (v/v/v). 20 mm Bis‐TRIS propane was used as buffer with 100 µm of 20:4(n‐6). Reaction happened at pH A: 8.0. B: 7.5. C: 7.0. D: 6.5. Chromatograms of CP‐HPLC in n‐hexane:isopropanol:TFA 100:2:0.1 (v/v/v) is given for all 4 HETEs obtained after reaction at pH 7.5 in each corresponding box. [file PLB-21-131-s003.pdf]

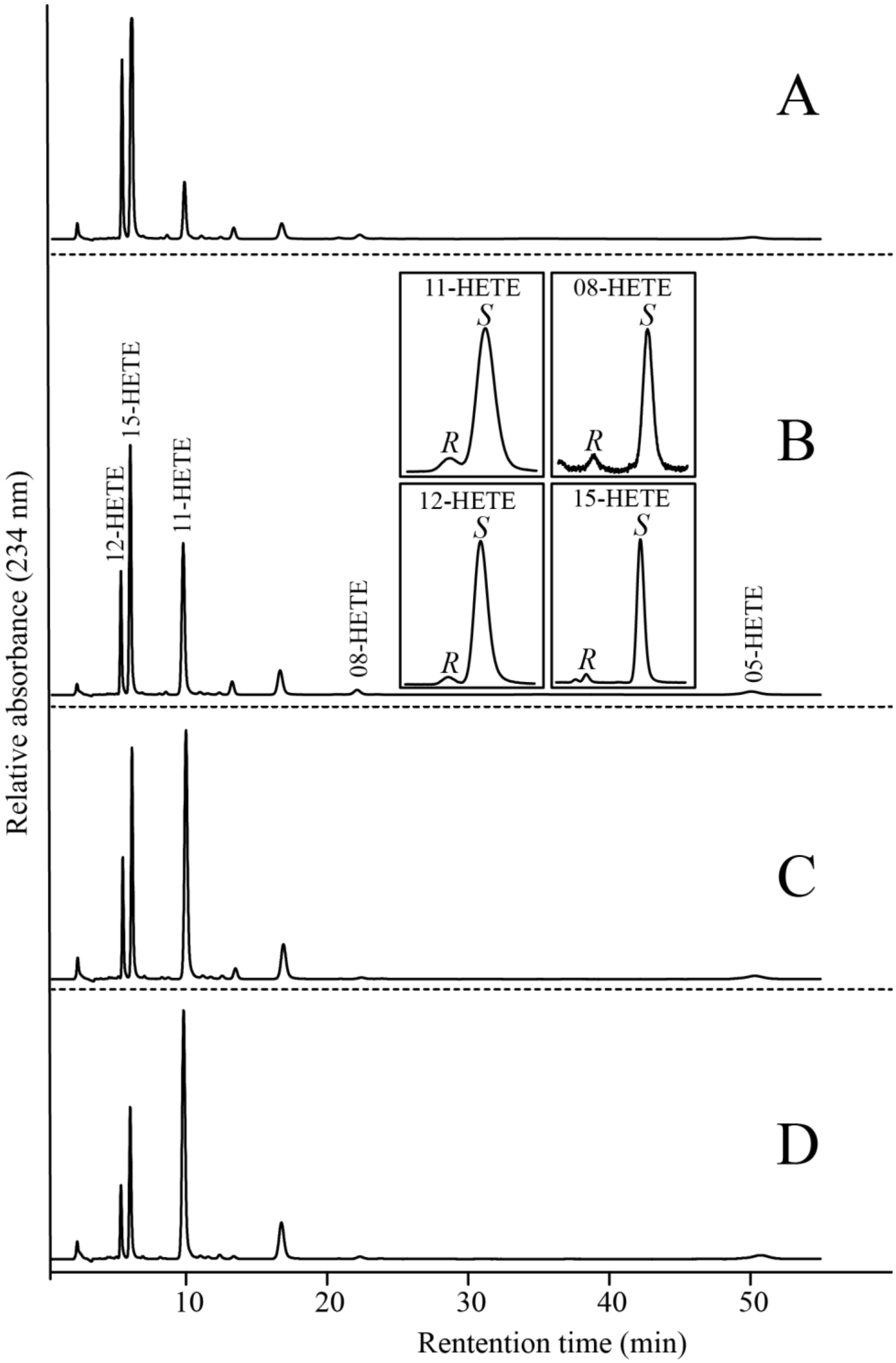

Additional file 3 Comparison of 20:4(n-6) oxidation products of LiLOX N702T/F703V mutant at different pH. **A-D.** Regioisomers separated by SP-HPLC in n-hexane:isopropanol:TFA 100:1:0.1 (v/v/v). 20 mM Bis-TRIS propane was used as buffer with 100 µM of 20:4 (n-6). Reaction happened at pH **A.** 8.0. **B.** 7.5. **C.** 7.0. **D.** 6.5. Chromatograms of CP-HPLC in n-hexane:isopropanol:TFA 100:2:0.1 (v/v/v) is given for all 4 HETEs obtained after reaction at pH7.5 in each corresponding box.
