# Supplementary material for: The green microalga Lobosphaera incisa harbours an arachidonate 15S‐lipoxygenase
Source: Plant Biol (Stuttg). 2018 Oct 24;21(Suppl Suppl 1):131–42. doi: 10.1111/plb.12920 (PMC6587457; doi:10.1111/plb.12920)
Supplement: Supplementary file 4 — File S4. Alignment of five conserved sequence motifs of LOXs. Residue numbers are those from the LiLOX sequence. The five amino acid residues responsible for iron binding are framed and located in the first, fourth and fifth motif. In addition, the second motif shows the arginine residue at position 689 that may interact with carboxylate residue from the fatty acid substrate and the third motif shows two residues that are located at the bottom of the substrate‐binding pocket (702 and 703). For every given residue, identity represents its homology between all 13S‐LOXs from Fig. 7 used in this alignment (Green = 100% < Gold < 50% ≤ Red). Allignment obtained from the software Geneious, Muscle allignment with default parameters. LOXs accession numbers are as indicated in Fig. 2. [file PLB-21-131-s004.pdf]

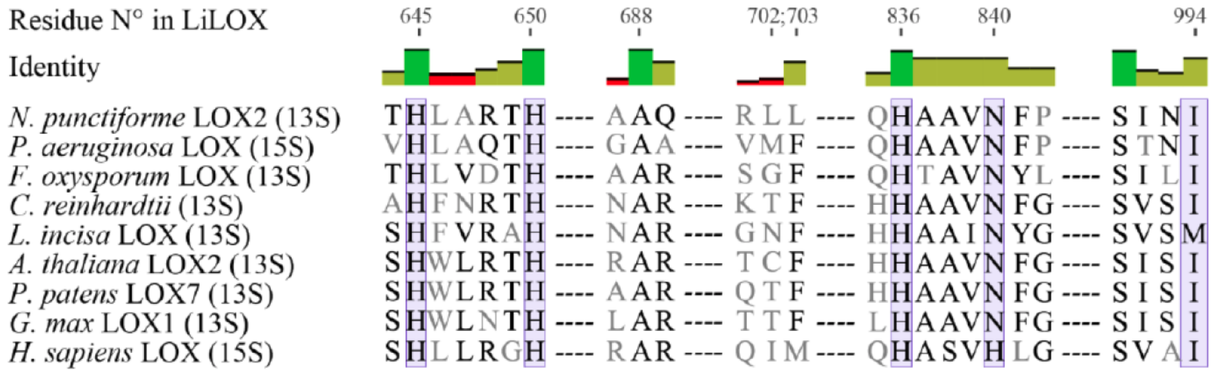

Additional file 4 Alignment of five conserved sequence motifs of LOXs. Residue numbers are those from the LiLOX sequence. The five amino acid residues responsible for iron binding are framed and located in the first, fourth and fifth motif. In addition, the second motif shows the arginine residue at position 689 that may interact with carboxylate residue from the fatty acid substrate and the third motif shows two residues that are located at the bottom of the substrate-binding pocket (702 and 703). For every given residue, identity represents its homology between all 13S-LOXs from Figure 7 used in this alignment (Green = 100 % < Gold < 50 % ≤ Red). Allignment obtained from the software Geneious, Muscle allignment with default parameters. LOXs accession numbers are as indicated in Fig. 2.
