# Supplementary material for: The green microalga Lobosphaera incisa harbours an arachidonate 15S‐lipoxygenase
Source: Plant Biol (Stuttg). 2018 Oct 24;21(Suppl Suppl 1):131–42. doi: 10.1111/plb.12920 (PMC6587457; doi:10.1111/plb.12920)
Supplement: Supplementary file 5 — File S5. Summary of the shares from regio‐ and stereoisomers, after oxidation by LiLOX and LiLOX mutants with PUFA 20:4(n‐6). All regioisomers from 20:4(n‐6) were separated by SP‐HPLC and integrated. Data represent the average of the percentage of each regioisomers, from three independent experiments, performed with different enzyme preparations. The regioisomers present in sufficient amount were collected and analyzed by CP‐HPLC to separate both stereoisomers. The share of S isomers are presented in percentage. Rac: racemic. [file PLB-21-131-s005.pdf]

Additional file 5 Summary of the shares from regio- and stereoisomers, after oxidation by LiLOX and LiLOX mutants with PUFA 20:4(n-6). All regioisomers from 20:4(n-6) were separated by SP-HPLC and integrated. Data represent the average of the percentage of each regioisomers, from three independent experiments, performed with different enzyme preparations. The regioisomers present in sufficient amount were collected and analysed by CP-HPLC to separate both stereoisomers. The share of *S* isomers are presented in percentage. Rac : racemic.

		12-HETE	*S*	15-HETE	*S*	11-HETE	*S*	8-HETE	*S*	5-HETE	*S*
**N702T/F703V**	**pH 6.5**	**8.9**		**23.9**	96.6	**61.7**	92.0	**1.8**		**3.7**	
	**pH 7.0**	**12.8**		**36.6**		**43.7**		**3.7**		**3.3**	
	**pH 7.5**	**14.1**	95.9	**49.9**	95.7	**28.0**	92.9	**5.1**	90.9	**2.9**	rac
	**pH 8.0**	**18.2**	93.0	**58.3**	89.5	**15.7**	80.0	**5.5**		**2.3**	
**N702T/F703V/ R853L**	**pH 6.5**	**14.1**		**50.0**	92.48	**27.0**	86.6	**6.4**		**2.5**	
	**pH 7.0**	**15.4**		**62.5**		**15.2**		**5.2**		**1.7**	
	**pH 7.5**	**15.8**	98.1	**69.0**	97.4	**9.7**	87.7	**4.4**	87.0	**1.1**	
	**pH 8.0**	**15.8**		**73.3**		**6.4**		**3.3**		**1.2**	
**N702T/F703V/ R853M**	**pH 6.5**	**12.0**		**36.8**	98.1	**39.8**	86.72	**10.3**		**1.1**	
	**pH 7.0**	**15.3**		**48.1**		**27.9**		**8.2**		**0.4**	
	**pH 7.5**	**16.0**	98.8	**60.5**	95.1	**15.4**	91.5	**7.2**	76.0	**0.9**	
	**pH 8.0**	**18.4**		**67.3**		**9.6**		**4.4**		**0.3**	
