# Supplementary material for: The green microalga Lobosphaera incisa harbours an arachidonate 15S‐lipoxygenase
Source: Plant Biol (Stuttg). 2018 Oct 24;21(Suppl Suppl 1):131–42. doi: 10.1111/plb.12920 (PMC6587457; doi:10.1111/plb.12920)
Supplement: Supplementary file 6 [file PLB-21-131-s006.docx]

**Supporting Information**

Additional Supporting Information may be found online in the supporting information tab for this article:

**Additional file 1** Shorter and longer versions of the putative LOX from *L. incisa*. The shorter version is shown in black. The longer version corresponds to the whole sequence, in red and black.

**Additional file 2** Identification of regioisomers and stereoisomers of LiLOX products. The reaction mixture contained 1 µg of purified LiLOX in 20 mM Bis-TRIS propane buffer pH 7.5 and 100 µM of respective PUFA. Reactions took place for 1 h at room temperature with accessible oxygen. Products were reduced by sodium borohydride for 10 minutes at room temperature and extracted with diethylether. **A-E.** SPHPLC chromatogram of LiLOX products derived from the following PUFA acid substrates: **A.** 18:2(n-6); **B.** 18:3(n-6); **C.**18:3(n-3); **D.** 20:4(n-6); **E.** 16:3(n-3). Results shown are representative for three independent experiments. Each experiment was performed with different enzyme preparations. For each main product, the respective chromatogram of the Chiral Phase CP‑HPLC is represented in the corresponding box, representative of one measurement. All isomers were identified with authentic standards besides the Stereoisomers 11*R*-HHTE/11*S*-HHTE and 11*R*-HHDE/11*S*-HHDE which were tentatively assigned. All LiLOX products were detected at 234 nm.

**Additional file 3** Comparison of 20:4(n-6) oxidation products of LiLOX N702T/F703V mutant at different pH. **A-D.** Regioisomers separated by SP-HPLC in n-hexane:isopropanol:TFA 100:1:0.1 (v/v/v). 20 mM Bis-TRIS propane was used as buffer with 100 µM of 20:4(n-6). Reaction happened at pH **A.** 8.0. **B.** 7.5. **C.** 7.0. **D.** 6.5. Chromatograms of CP-HPLC in n-hexane:isopropanol:TFA 100:2:0.1 (v/v/v) is given for all 4 HETEs obtained after reaction at pH7.5 in each corresponding box.

**Additional file 4** Alignment of five conserved sequence motifs of LOXs. Residue numbers are those from the LiLOX sequence. The five amino acid residues responsible for iron binding are framed and located in the first, fourth and fifth motif. In addition, the second motif shows the arginine residue at position 689 that may interact with carboxylate residue from the fatty acid substrate and the third motif shows two residues that are located at the bottom of the substrate-binding pocket (702 and 703). For every given residue, identity represents its homology between all 13S-LOXs from Figure 7 used in this alignment (Green = 100 % < Gold < 50 % ≤ Red). Allignment obtained from the software Geneious, Muscle allignment with default parameters. LOXs accession numbers are as indicated in Fig. 2.

**Additional file 5** Summary of the shares from regio- and stereoisomers, after oxidation by LiLOX and LiLOX mutants with PUFA 20:4(n-6). All regioisomers from 20:4(n-6) were separated by SP-HPLC and integrated. Data represent the average of the percentage of each regioisomers, from three independent experiments, performed with different enzyme preparations. The regioisomers present in sufficient amount were collected and analyzed by CP-HPLC to separate both stereoisomers. The share of *S* isomers are presented in percentage. Rac : racemic.
